# Screening of exopolysaccharide-producing *Enterobacter aerogenes* NJ1023 and its cadaverine biosynthesis promotion

**DOI:** 10.3389/fmicb.2023.1200123

**Published:** 2023-07-27

**Authors:** Yanai Xie, Zhen Ye, Xin Wan, Hua Deng, Weihao Sun, Xun He, Kequan Chen

**Affiliations:** State Key Laboratory of Materials Oriented Chemical Engineering, College of Biotechnology and Pharmaceutical, Nanjing Tech University, Nanjing, China

**Keywords:** *Enterobacter aerogenes*, extracellular polysaccharides, EPSs, cytoprotection, cadaverine

## Abstract

*Enterobacter aerogenes*, the gram-negative bacteria belonging to the family *Enterobacteriaceae*, lacks the ability to synthesize chemicals. However, in this study, a strain of *Enterobacter aerogenes* NJ1023 screened from the soil containing petrochemicals was found to be capable of producing extracellular polysaccharides (EPSs). After purification of the polysaccharide, the chemical composition and physicochemical properties of the polysaccharide were analyzed by UV–Vis spectra, FTIR spectroscopy and GC-MS, etc. The results showed that: The molecular weight of the polysaccharide produced by this strain was only 2.7×10^3^ Da, which was lower than that reported in other polysaccharides from the same genus. The polysaccharide produced by *E. aerogenes* NJ1023 mainly comprised xylose, glucose, galactose, and N-acetylglucosamine with a molar ratio of 0.27: 4.52: 1.74: 0.2, which differed from those reported from the same genus. The results demonstrated that lower incubation temperatures and shaking speeds were more favorable for EPSs synthesis, while higher incubation temperatures and shaking speeds favored cell growth. Additionally, the EPSs produced by *E. aerogenes* NJ1023 significantly protected the *Escherichia coli* cells against cadaverine stress. Overall, the discovery of EPSs produced by *E. aerogenes* increased the diversity of bacterial polysaccharides and broadened the potential applications of this species.

## 1. Introduction

Extracellular polysaccharides (EPSs) are the secondary metabolites produced during bacterial fermentation, which can either remain attached to the cell surface or be excreted into the growth medium (Wear et al., 2022). These polysaccharides play significant roles in protecting cells against adverse environments, intercellular aggregation, surface adhesion, and biofilm formation (Butorac et al., 2021). Additionally, EPSs possess versatile properties and functionalities, making them intriguing microbial products for developing nutraceuticals, chemical auxiliaries, and flexible materials (Asaki et al., 2020; Harling et al., 2020; Li et al., 2022).

The EPS-producing strains include the representatives of *Bacillus*, lactic acid bacteria (LAB), and *Enterobacter* groups (Zhao et al., 2017; Asgher et al., 2020; Abdalla et al., 2021). The EPSs from these microorganisms have diverse structural and physicochemical properties due to different originating strains, culture conditions, and substrate composition (Jaroszuk-Ściseł et al., 2020). Zhu et al. (2018) screened a *Bacillus atrophaeus* WYZ strain isolated from mangroves with an extracellular polysaccharide yield of 580 mg/L. The synthesized polysaccharide was composed of glucose, rhamnose, salicin, and glucuronic acid with a molar ratio of 84.4: 7.2: 6.7: 1.7 and a porous twisted three-dimensional cobweb structure with high water retention and permeability. Cao et al. (2020) isolated a *Bacillus velezensis* SN − 1 strain from naturally fermented barley with an extracellular polysaccharide yield of 2.7 g/L using sucrose as the sole carbon source. The synthesized polysaccharide was composed of glucose, mannose, and fructose, with high thermal stability and free radical scavenging ability. Similarly, Asgher et al. (2020) obtained a mutant strain *Bacillus licheniformis* MS3 using short wavelength ultraviolet (UV) mutagenesis, with an extracellular polysaccharide yield of 15.6 g/L during fermentation. This polysaccharide was a heteropolysaccharide (HePS) composed of glucose (46.80%), fructose (32.58%), and mannose (20.60%), with high hydrophilic and lipophilic properties. LAB representing several genera (*Lactobacillus* spp., *Lactococcus* spp., *Streptococcus* spp., *Lactococcus lamellus*, *Clostridium lucidum*, and *Bifidobacterium*) could produce structurally diverse EPSs with valuable functional properties and potential applications (Peteris et al., 2020). LAB has the potential to synthesize HePs containing repeating units of different monosaccharides and non-sugar molecules and produce homopolysaccharides (HoPS) containing only one monosaccharide (glucose or fructose) (Costa et al., 2020). The EPSs synthesized by *Lb. fermentum* spp. and *Lb. delbrueckii* spp. mainly consisted glucose and galactose backbones, while the EPSs secreted by *Lactobacillus* KC117496 consisted glucans, the glucose polymers (Thi et al., 2020). However, in most cases, the total amount of extracellular polysaccharides synthesized by LAB did not exceed 1 g/L even under optimal growth conditions. Only some *Lactobacillus* and *Streptococcus* exhibited higher EPSs yields (1.2–4.8 g/L). This relatively low concentration of EPSs has significantly hindered or even prevented the commercial availability of LAB species to produce higher yields of industrially important polysaccharides (Ozlem, 2015; Peteris et al., 2020).

Among *Enterobacteria*, only *Enterobacter cloacae* has been reported to produce EPSs. Isobe et al. (2001) isolated an *Enterobacter cloacae* strain from pond water, which synthesized EPSs using sucrose as the sole carbon source. The synthesized polysaccharides were composed of fucose, galactose, glucose, and glucuronide with a molar ratio of 2:3:2:1. Similarly, Iyer et al. (2005) screened an *E. cloacae* strain from marine sediments, and its EPSs were also composed of fucose, galactose, glucose, and glucuronide, with a molar ratio of 2:1:1:1. Wang et al. (2013) obtained EPSs composed of rockulose, glucose, galactose, glucuronide, and pyruvate with a molar ratio of 2:1:3:1:1 from extracellular metabolites of *E. cloacae*. *Enterobacter aerogenes*, has previously been regarded as a clinically significant bacterial pathogen (Davin-Regli and PagÃ, 2015). However, recently, *E. aerogenes* has been reported to produce various biochemical products, including biofuels (e.g., biohydrogen, bioethanol) (Richen et al., 2020; Fatemeh and Khosrow, 2021; Sawasdee et al., 2021), polyols (e.g., 1,4-butanediol) (Laxmi et al., 2019), organic acids (e.g., butanedioic acid) (Szczerba et al., 2020a,b), and enzymes (e.g., phytase) (Muslim et al., 2018). The present study aimed to investigate the ability of *E. aerogenes* obtained from soils contaminated with petrochemicals to produce EPSs and to evaluate its potential to improve substrate/product tolerance and catalytic efficiency under a hyperosmotic environment. Overall, this study reported a novel bacterial polysaccharide as a cytoprotective agent, expanding the application of polysaccharide science.

## 2. Materials and methods

### 2.1. Chemicals

All soil samples were collected near the Yangzi Petrochemical Plant (Nanjing, China), and the resultant bacterial isolates were deposited into the laboratory’s collection. Standard monosaccharides and trifluoroacetic acid (TFA) were purchased from Sigma Chemicals Co., Ltd. (St. Louis, United States). All other chemicals and reagents were of reagent grade and purchased from the China National Pharmaceutical Group.

### 2.2. Screening of EPSs producing bacteria

The soil samples were suspended at 20% (w/v) in sterilized water to isolate the EPS-producing bacteria, and then the suspension was filtered to remove the soil. The filtrate was added to the Nutrient Broth Medium (Toupu Biol-engineering Co., Ltd., Zhaoyuan, China) at a ratio of 1: 9 and incubated at 30°C for 48 h. The culture was then diluted (10^−1^ to 10^−6^) with sterilized water. From each dilution, 200 μL was plated onto solid nutrient agar plates and incubated at 30°C for 48 h. Single colonies were selected for Gram staining and microscope (XS-212-202, Japan) observation and inoculated into the fermentation medium. The fermentation medium (3.0 g/L beef extract, 10.0 g/L peptone, 2.0 g/L yeast extract, 5.0 g/L NaCl, pH 7.4–7.6) was used to produce EPSs with shaking (250 rpm) for 72 h at 30°C. The strain with the highest EPSs yield was used as the test strain, and subsequent genetic identification of this strain based on 16S rDNA sequencing was performed by Paiseno Biological Co., LTD (Shanghai, China). The observed bacterial growth index was evaluated using dry cell weight (DCW) and EPSs yield. The DCW was computed from a curve of OD_600_ with respect to dry weight. An OD_600_ of 1.0 represented 400 mg dry weight/L. The EPSs yield was defined as EPSs production per unit of DCW.

### 2.3. Exopolysaccharide extraction and purification

Crude EPSs were isolated from the fermentation medium using improved methods reported by Sun et al. (2021). The culture broth was diluted with ultra-pure water, mixed with diatomite, and filtered by vacuum filtration. The filtrate was concentrated to 1/4 of the initial volume. Later, the concentrated supernatant was deproteinated by washing thrice with Sevag reagent (isoamyl alcohol: chloroform = 1:4, v/v). Before collection, the crude EPSs were precipitated with ethanol (1:5, v/v) at 4°C overnight. The EPSs yield was evaluated after lyophilization. EPSs purification was performed according to the previously described method (Sun et al., 2021). The crude EPSs were dissolved in deionized water, centrifuged (15,000 × *g*, 30 min), and the supernatant was applied to a DEAE-cellulose DE-32 column (16 mm × 40 cm) (Shanghai yuanye Bio-Technology Co Ltd., China) equilibrated with 0.1 M PBS buffer (pH 7.0). The column was sequentially eluted with 200 mL deionized water and 200 mL NaCl solutions at different concentrations of 0.3, 0.5, 0.7, 1.0 mol/L and a flow rate of 1 mL/min, respectively. The eluents (10 mL/tube) were monitored by the phenol-sulfuric acid method. The fractions obtained by 0.5 mol/L and 0.7 mol/L NaCl were collected and further purified by gel permeation chromatography on a Sephadex G-25 column (16 mm × 40 cm) using ultra-pure water as an eluent at a flow rate of 0.2 mL/min. The fractions were collected, concentrated and lyophilized, and then the pure polysaccharide was obtained.

### 2.4. Exopolysaccharide analysis

The UV–Vis spectra, FTIR spectroscopy, average molecular weight, chemical composition, and uronic acid analysis of EPSs were performed according to the previously reported methods (Sun et al., 2021). The surface charge of a polysaccharide was determined using Zeta potential measurement according to the method reported by Varma and Kumar (2018). EPSs derivatization was performed following a previously reported method (Wang et al., 2018), and the operating conditions for monosaccharide composition analysis were set as mentioned in a previous method (Tayebeh et al., 2019). Monosaccharides were identified by comparing them with standard glucose, galactose, xylose, and N-acetylglucosamine.

### 2.5. Effects of exopolysaccharides on the whole-cell synthesis of cadaverine

Whole-cell synthesis of cadaveric amines was performed in a 1 L fermenter (Infors, Switzerland) with a conversion fluid volume of 0.3 L. The transformation broth contained 50 g/L L-lysine and 3 g DCW/L of engineered *Escherichia coli* DFC1001 (Guo et al., 2020) with or without EPSs. Approximately 100–300 mg of EPSs were added per gram of DCW *E. coli* DFC1001. Biotransformation was performed at 37°C, 300 rpm, and the pH was adjusted to 7.0 by the automatic addition of 2 M HCl. After 1 h of biotransformation, the surviving bacteria in the culture solution were calculated by plating on Luria-Bertani (LB) agar plates containing 50 μg/mL streptomycin (Guo et al., 2020), followed by serial dilution. The survival rate of engineered *Escherichia coli* DFC1001 was defined as the percentage of viable cells after and before whole-cell synthesis. The cadaverine yield was calculated by dividing the cadaverine concentration by the initial concentration of the substrate L-lysine (50 g/L). The culture solution was centrifuged at 4000 x *g* for 5 min and the concentration of cadaverine was determined by high-performance liquid chromatography. Cadaverine was separated using a YMC Carotenoid C30 column (4.6 × 250 mm, 5 μm). The values of flow rate, injection volume, and column temperature were set at 0.8 mL/min, 10 μL, and 35°C, respectively. 0.5% trifluoroacetic acid and 5% acetonitrile were used as mobile phases A and B, respectively.

### 2.6. Factors affecting EPSs synthesis

Single-factor tests were employed to investigate the effects of fermentation conditions on bacterial growth and EPSs synthesis. The strains were inoculated in the fermentation medium and cultured in a shaker incubator. The range of fermentation conditions (influencing factors and levels) were selected as follows: pH (5.02, 5.86, 7.13, 8.19, 8.61), rotational speed (0, 50, 100, 150, 200, 250 rpm), temperature (10, 18, 24, 30, 37°C). Then, the preliminary range of synthetic variables, namely pH (X_1_), rotational speed (X_2_), and temperature (X_3_), was determined based on single factor experiments. A three-factor-three level Box–Behnken design was employed using design export software to determine which combination of synthesis variables would yield the largest amount of dry cell weight (DCW) (Y_1_) and EPSs field (Y_2_).

## 3. Results and discussion

### 3.1. Isolation and identification of EPSs-producing strains

About 54 single colonies with the EPSs producing ability were isolated from the soil samples. These 54 strains were cultured and fermented separately using the same medium and growth conditions. EPSs production by different strains was measured, as shown in Table 1. The strain numbered NJ 1023 showed the highest EPSs yield.

**Table 1:** Polysaccharide-producing strains isolated from soil samples contaminated with petrochemicals.

Strains	Gram	EPSs yield (mg/g DCW)	Strains	Gram	EPSs yield (mg/g DCW)
NJ 1001	G^+^	0.12 ± 0.02	NJ 1028	G^+^	60.01 ± 2.45
NJ 1002	G^+^	0.01 ± 0.01	NJ 1029	G^+^	1.16 ± 0.22
NJ 1003	G^+^	0.44 ± 0.12	NJ 1030	G^+^	2.09 ± 0.12
NJ 1004	G^−^	2.56 ± 0.32	NJ 1031	G^+^	1.65 ± 0.17
NJ 1005	G^−^	37.38 ± 1.14	NJ 1032	G^+^	12.43 ± 0.22
NJ 1006	G^+^	1.34 ± 0.02	NJ 1033	G^+^	1.66 ± 0.09
NJ 1007	G^+^	39.66 ± 1.56	NJ 1034	G^−^	32.11 ± 2.13
NJ 1008	G^+^	1.19 ± 0.11	NJ 1035	G^+^	2.78 ± 0.03
NJ 1009	G^−^	0.09 ± 0.02	NJ 1036	G^−^	64.45 ± 1.58
NJ 1010	G^−^	1.34 ± 0.01	NJ 1037	G^+^	2.08 ± 0.16
NJ 1011	G^+^	0.51 ± 0.02	NJ 1038	G^−^	1.08 ± 0.02
NJ 1012	G^−^	1.48 ± 0.22	NJ 1039	G^−^	61.78 ± 1.10
NJ 1013	G^+^	28.32 ± 0.89	NJ 1040	G^+^	1.01 ± 0.01
NJ 1014	G^−^	44.68 ± 2.55	NJ 1041	G^+^	2.16 ± 0.02
NJ 1015	G^−^	0.06 ± 0.01	NJ 1042	G^+^	2.04 ± 0.02
NJ 1016	G^−^	0.06 ± 0.01	NJ 1043	G^−^	1.06 ± 0.12
NJ 1017	G^+^	1.44 ± 0.12	NJ 1044	G^+^	41.69 ± 0.45
NJ 1018	G^−^	1.19 ± 0.13	NJ 1045	G^+^	1.16 ± 0.02
NJ 1019	G^−^	1.09 ± 0.01	NJ 1046	G^−^	0.57 ± 0.03
NJ 1020	G^+^	0.05 ± 0.01	NJ 1047	G^+^	1.16 ± 0.12
NJ 1021	G^+^	0.06 ± 0.01	NJ 1048	G^−^	1.23 ± 0.16
NJ 1022	G^−^	0.57 ± 0.02	NJ 1049	G^−^	2.18 ± 0.33
NJ 1023	G^−^	70.62 ± 1.24	NJ 1050	G^+^	2.09 ± 0.15
NJ 1024	G^−^	0.02 ± 0.01	NJ 1051	G^+^	59.63 ± 2.19
NJ 1025	G^+^	0.06 ± 0.02	NJ 1052	G^+^	1.98 ± 0.17
NJ 1026	G^+^	0.06 ± 0.03	NJ 1053	G^−^	2.33 ± 0.02
NJ 1027	G^+^	0.09 ± 0.03	NJ 1054	G^+^	2.06 ± 0.14

The 16S rDNA nucleotide sequence of strain NJ1023 was analyzed by BLAST software. Strain NJ1023 had 99.79% 16S rDNA similarity with its closest relative *E. aerogenes* B19. Therefore, strain NJ1023 was identified as an *E. aerogenes* and was submitted to the China Center for Type Culture Collection (CCTCC) (Wuhan, China) with an accession number of CCTCC M 20211243.

### 3.2. Characteristics of EPSs

**Figure 1:**
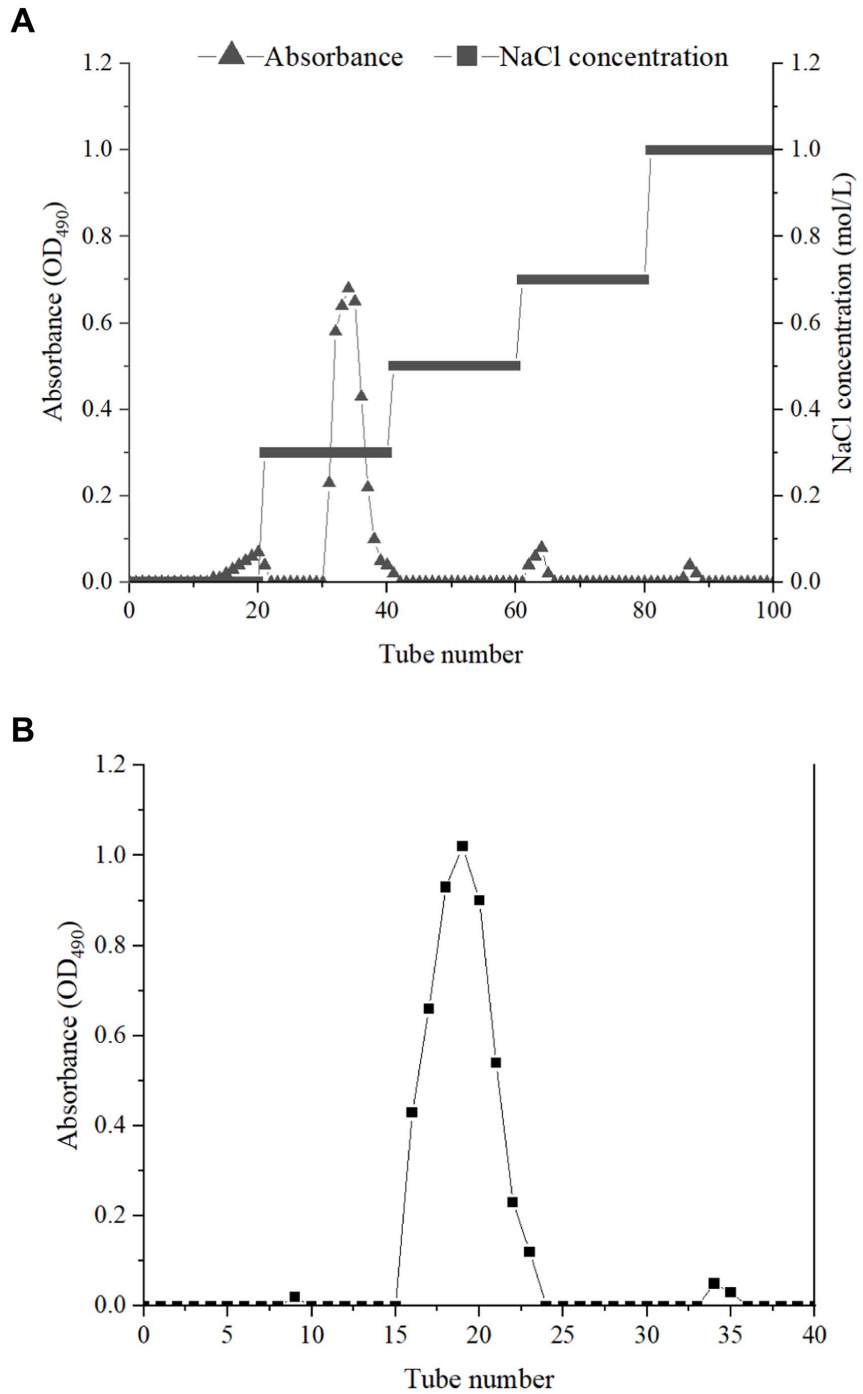
Elution curves of crude EPSs on DEAE-cellulose DE-32 column **(A)** and Sephadex G-25 column **(B)**.

The crude EPSs were extracted from *E. aerogenes* NJ1023 by vacuum filtration, deproteinization, ethanol precipitation, and lyophilization. The dry sample was then separated by DEAE-cellulose DE-32, and one fraction was obtained (Figure 1A). The fraction eluted with 0.3 M NaCl solutions was further purified with a Sephadex G-25 size-exclusion chromatography column. The major elution fraction (Figure 1B) was collected. The EPSs produced by *E. aerogenes* NJ1023 contained approximately 92.44% total sugar, 0.41% protein, 3.40% glycuronic acid, 2.13% P, and 0.85% S. The absorption peaks were not found at 260 nm, indicating an absence of nucleic acids. A novel molecular weight of 2.7 × 10^3^ Da was calculated using known MW standards. Complete hydrolysis of the EPSs, followed by gas chromatography mass spectrometry (GC–MS) demonstrated that the polysaccharide was composed of D-xylose, D-glucose, D-galactose, and N-acetylglucosamine with a molar ratio of 0.27: 4.52: 1.74: 0.2. The molecular weight and monosaccharide composition of these EPSs significantly differed from the EPSs produced by other strains of the same genus. EPSs produced by other strains of *Enterobacter* spp. had a high molecular weight of approximately 1.0 × 10^6^ Da, and were mainly composed of fucose, galactose, and glucose (Isobe et al., 2001; Wang et al., 2013).

**Figure 2:**
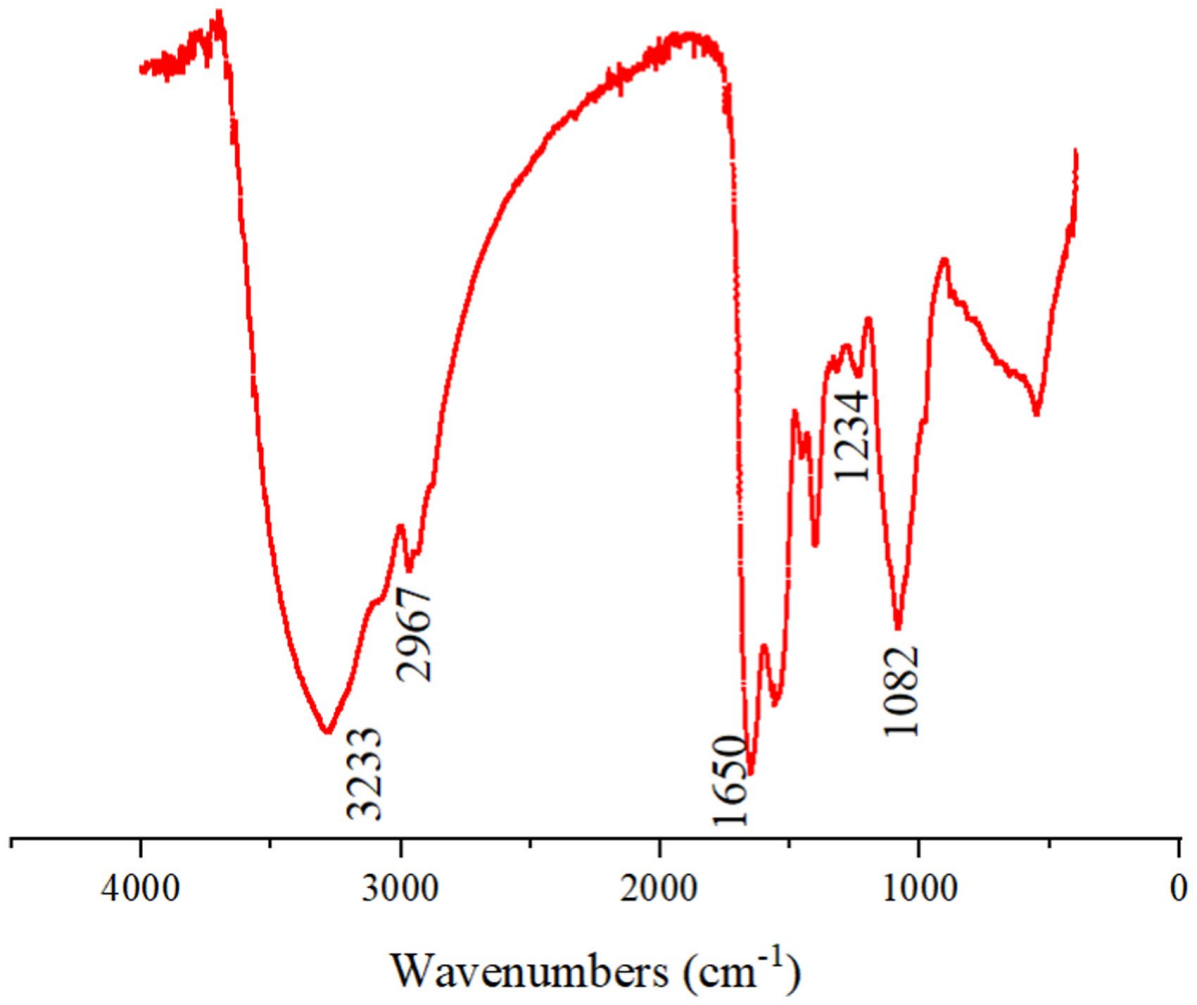
Infrared spectrum of EPSs from *Enterobacter aerogenes* NJ1023.

In the infrared wavelengths, the EPSs had the characteristics of polysaccharides (Figure 2). The sample showed the absorption peaks characteristic of polysaccharides at 3233, 2967, 1,650, 1,234, and 1,082 cm^−1^. The peaks near 3,233 cm^−1^ were attributed to the stretching vibration of O-H. Low intensity banding at 2967 cm^−1^ was attributed to the C-H stretching and bending. The peak observed at 1650 cm^−1^ was attributed to the C=O stretch of amide. The absorption band at 1234 cm^−1^ was attributed to the sugar structure of O-H in the carboxyl group, indicating the presence of glycuronic acid in the polysaccharide (Guo et al., 2022).

### 3.3. Effects of EPSs on whole-cell synthesis of cadaverine

Cadaverine, a common polyamine with important physiological functions, plays a crucial role in maintaining cell morphology and physiological functions (Igarashi and Kashiwagi, 2018). However, excessive polyamines can be toxic to cells, causing oxidative damage (Murray et al., 2018). Polysaccharides are often used as antioxidants to significantly reduce oxidation and protect cells (Li et al., 2020). This study aimed to investigate the role of EPSs from *E. aerogenes* NJ1023 in response to cadaverine toxicity in *E. coli* DFC1001 cells. As shown in Table 2, the addition of extracellular polysaccharides increased the conversion of lysine to cadaverine, which could be attributed to the increased survival rate of the engineered *E. coli* cells. Compared to the control without EPSs addition, the addition of 300 mg/g EPSs increased the survival rate of the strain by 57.27%.

**Table 2:** EPSs protect engineered *E. coli* cells from cadaverine toxicity.

	Survival rate (%)	Cadaverine yield (%)
No EPSs added	50.01 ± 3.56	23.31 ± 1.13
EPSs added		
100 mg/g	62.33 ± 4.67	27.54 ± 3.14
200 mg/g	70.88 ± 2.78	32.88 ± 2.52
250 mg/g	73.19 ± 2.12	35.49 ± 2.02
300 mg/g	78.65 ± 3.00	38.77 ± 1.41

### 3.4. Optimization of EPSs synthesis conditions

#### 3.4.1. pH

**Figure 3:**
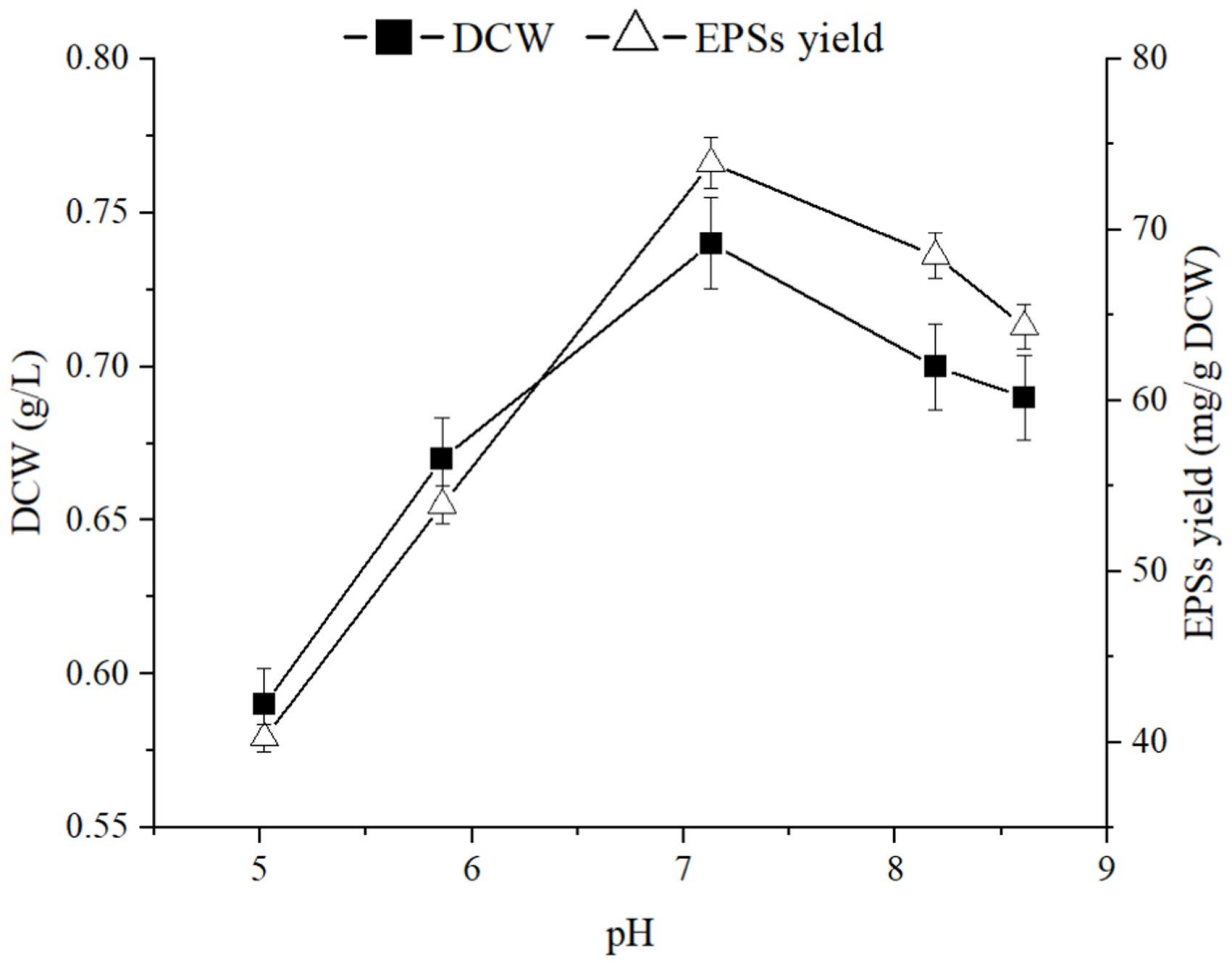
Effects of pH on cell growth and polysaccharide production of *E. aerogenes* NJ1023.

Culture conditions have significant impacts on cell growth and the extracellular polysaccharide synthesis capacity of microorganisms (Yang et al., 2022). The fermentation processes were performed under the pH range of 5.02 to 8.61 to investigate the impact of pH on EPSs synthesis and cell growth. Fermentation temperature and rotational speed were set at 30°C and 220 rpm, respectively. As shown in Figure 3, when the pH was 7.13, the highest sugar production and cell growth were 83.95 mg/g and 0.72 g/L, respectively. Therefore, a pH of approximately 7 was most favorable for biomass production and polysaccharide accumulation in this strain.

#### 3.4.2. Rotational speed

**Figure 4:**
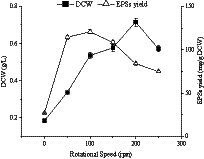
Effects of rotational speed on cell growth and polysaccharide production of *E. aerogenes* NJ1023.

The rotational speed of the fermentation process had different effects on cell growth and extracellular polysaccharide synthesis of *E. aerogenes* NJ1023 (Figure 4). During fermentation, the ammonium hydroxide solution (6 M) was automatically added to maintain a pH of 7, and the fermentation temperature was set at 30°C. As shown in Figure 4, the highest cell growth (0.72 g/L) was observed for *E. aerogenes* NJ1023 at a rotational speed of 200 rpm. However, optimal polysaccharide synthesis of 120.62 mg/g DCW was obtained at a rotational speed of 100 rpm. Therefore, a lower rotational speed was more suitable for *E. aerogenes* NJ1023 to accumulate EPSs, while a higher rotational speed was more suitable for cell proliferation. This result provides novel prospects for regulating the fermentation conditions of this strain in the future.

#### 3.4.3. Temperature

**Figure 5:**
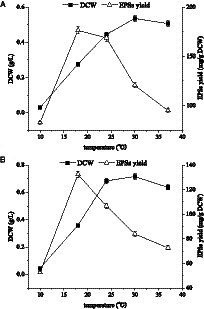
Effects of culture temperature on cell growth and polysaccharide production of *E. aerogenes* NJ1023. **(A)** At a rotational speed of 100 rpm. **(B)** At a rotational speed of 200 rpm.

In this study, the effects of temperature on cell growth and polysaccharide yield of *E. aerogenes* NJ1023 were investigated under the previously established optimum rotational speed (100 rpm) for sugar production and the optimum speed (200 rpm) for cell growth, respectively. As shown in Figure 5, when the temperature was 18°C and the rotational speed was 100 rpm, the optimal EPS yield was 176.45 mg/g (Figure 5A). The cell growth reached its maximum level (0.72 g/L) when the fermentation temperature was 30°C, and the rotational speed was 200 rpm (Figure 5B). These findings indicated that temperature had a significant impact on the growth and yield of *E. aerogenes* NJ1023, with lower temperatures being suitable for polysaccharide accumulation and higher temperatures more suitable for cell growth.

#### 3.4.4. Response surface methodology (RSM)

The fermentation conditions of *Enterobacter aerogenes* NJ1023 were optimized with response surface methodology (RSM). Based on the single-factor experimental tests, the pH (X_1_), rotational speed (X_2_), and temperature (X_3_) were selected as independent variables, and DCW (Y_1_) and EPSs yield (Y_2_) were used as response values, respectively, to design a three-factor, three-level experiment. The experimental factors and levels are shown in Table 3, and the experimental design and the results of the response surface are shown in Table 4. Regression analysis was performed on the test data, as shown in Table 4, and the multiple quadratic regression equation obtained was as follows (eqs 1, 2):

**Table 3:** Experimental factors and levels.

Levels	Factors		
	X_1_: pH	X_2_: rotational speed (rpm)	X_3_: temperature (°C)
DCW (Y_1_) as response values:			
−1	6.5	150	25
0	7.0	200	30
1	7.5	250	35
EPSs yield (Y_2_) as response values:			
−1	6.5	50	14
0	7.0	100	18
1	7.5	150	22

**Table 4:** Experimental design and results of response surface.

Run	X_1_	X_2_ (rpm)	X_3_ (°C)	Y_1_ (g/L)	Y_2_ (mg/g DCW)
1	0	−1	−1	0.39	95.55
2	−1	0	1	0.61	147.11
3	0	0	0	0.72	176.45
4	0	−1	1	0.58	140.14
5	−1	0	-1	0.41	98.52
6	0	0	0	0.70	175.33
7	0	0	0	0.72	177.62
8	0	0	0	0.72	176.35
9	0	1	−1	0.38	90.88
10	−1	1	0	0.62	152.11
11	1	1	0	0.65	157.61
12	1	0	1	0.54	146.44
13	1	−1	0	0.68	166.33
14	−1	−1	0	0.66	159.58
15	0	0	0	0.72	176.29
16	1	0	−1	0.48	105.33
17	0	1	1	0.50	132.78


(1) $$\begin{array}{l} Y_{1}=0.7160+0.0063X_{1}-0.0200X_{2}+0.0713X_{3}\\ \;\;\;\;\;\;+0.0025X_{1}X_{2}-0.0350X_{1}X_{3}-0.0175X_{2}X_{3}\\ \;\;\;\;\;\;-0.0080X_{1}{}^{2}\;-0.0555X_{2}{}^{2}-0.1980X_{3}{}^{2} \end{array}$$

(2) $$\begin{array}{l} Y_{2}=176.41+2.30X_{1}-3.53X_{2}+22.02X_{3}\\ \;\;\;\;\;\;\;-0.3125X_{1}X_{2}-1.87X_{1}X_{3}-0.6725X_{2}X_{3}\\ \;\;\;\;\;\;\;-3.99X_{1}{}^{2}-13.51X_{2}{}^{2}-48.06X_{3}{}^{2} \end{array}$$

The ANOVA results (Table 5) demonstrated that the above 2 quadratic regression models differed significantly (*p* < 0.01), indicating that the models fit similarly to the actual experiments and could better reflect the relationship between DCW and EPSs yield and each factor. When DCW was used as the response value, the interaction term X_2_X_3_ had a significant effect on the results (*p* < 0.05), the primary term X_2_ had a highly significant effect on the results (*p* < 0.01), and the primary term X_3_, the interaction term X_1_X_3_, the secondary term X_2_^2^ and X_3_^2^ had a very highly significant effect on the results (*p* < 0.001). When the EPSs yield was used as the response value, the interaction term X_1_X_3_ had a significant effect on the results (*p* < 0.05), the primary term X_1_ had a highly significant effect on the results (*p* < 0.01), and the primary term X_2_ and X_3_, the secondary term X_1_^2^, X_2_^2^ and X_3_^2^ had a very highly significant effect on the results (*p* < 0.001). The contribution of factors to the values of DCW and EPSs yield obtained by the *F*-test was as follows: temperature (X_3_) > rotational speed (X_2_) > pH (X_1_).

**Table 5:** Variance analysis of response surface experiments results.

Source	Sum of squares (Y_1_/Y_2_)	Df (Y_1_/Y_2_)	Mean square (Y_1_/Y_2_)	*F*-value (Y_1_/Y_2_)	*p*-value (Y_1_/Y_2_)
Model	0.2358/15083.93	9/9	0.0262/1675.99	184.29/1089.54	< 0.0001/< 0.0001
A	0.0003/42.27	1/1	0.0003/42.27	2.20/27.48	0.1817/0.0012
B	0.0032/99.55	1/1	0.0032/99.55	22.51/64.71	0.0021/< 0.0001
C	0.0406/3880.36	1/1	0.0406/3880.36	285.72/2522.57	< 0.0001/< 0.0001
AB	0.0000/0.3906	1/1	0.0000/0.3906	0.1759/0.2539	0.6875/0.6298
AC	0.0049/13.99	1/1	0.0049/13.99	34.47/9.09	0.0006/0.0195
BC	0.0012/1.81	1/1	0.0012/1.81	8.62/1.18	0.0218/0.3141
A^2^	0.0003/67.17	1/1	0.0003/67.17	1.90/43.66	0.2110/0.0003
B^2^	0.0130/768.11	1/1	0.0130/768.11	91.24/499.34	< 0.0001/< 0.0001
C^2^	0.1651/9726.94	1/1	0.1651/9726.94	1161.29/6323.35	< 0.0001/< 0.0001
Residual	0.0010/10.77	7/7	0.0001/1.54		
Lack of Fit	0.0007/8.12	3/3	0.0002/2.71	2.81/4.08	0.1717/0.1038
Pure Error	0.0003/2.65	4/4	0.0001/0.6625		
Cor Total	0.2368/15094.70	16/16			

**Figure 6:**
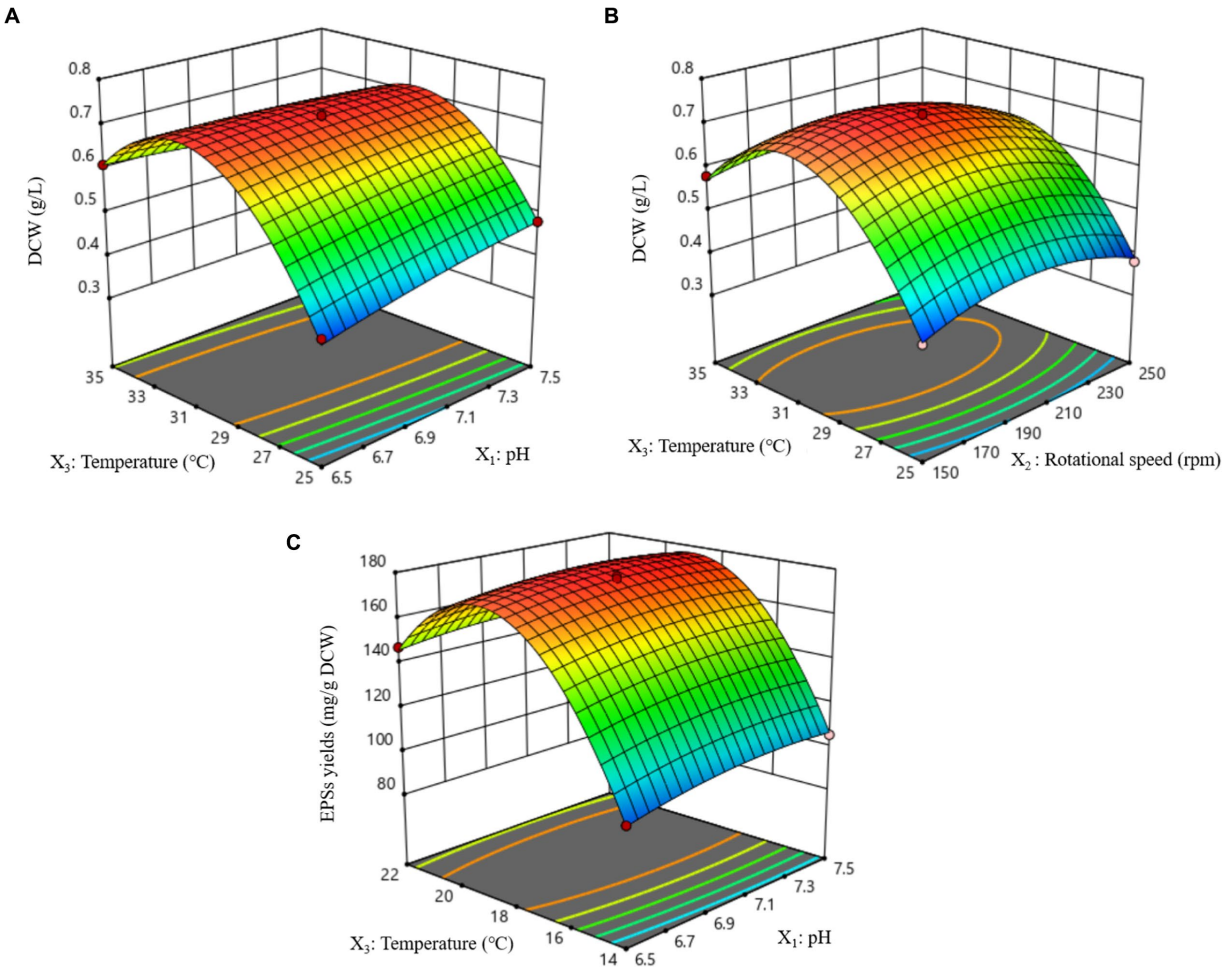
Response surface plots of effects of interaction between each factor on DCW and EPSs yield. **(A)** Effect of pH and temperature interaction on DCW. **(B)** Effect of rotational speed and temperature interaction on DCW. **(C)** Effect of pH and temperature interaction on EPSs yields.

The 3D response surfaces of the interactions of pH, rotational speed, and temperature on DCW and EPSs yield are shown in Figure 6. All figures generally show a trend of first rising and then falling. After the fitting equation, the optimum conditions for DCW are as follows: pH, 7,0; rotational speed, 189 rpm; temperature, 31°C. Accordingly, the theoretical highest DCW was predicted as 0.73 g/L at the optimum conditions. Verification experiments were performed for three replicates, and the average yield of DCW was 0.74 g/L. These experimental results were in good agreement with the predicted values by the model. The optimal experimental scheme for EPSs synthesis was obtained as follows: when the pH, rotational speed, and temperature were 7.12, 93.05 rpm, and 18.90°C, respectively, the EPSs yield reached as high as 179.41 mg/g DCW. Verification experiments were performed for three replicates, and the average yield of EPSs was 179.82 mg/g DCW. These experimental results were in good agreement with the predicted values by the model.

## 4. Conclusion

In summary, a *E. aerogenes* NJ1023 strain capable of synthesizing EPSs was obtained from petrochemical contaminated soil. To the best of our knowledge, this is the first report to highlight the EPSs synthesis property of *E. aerogenes*. Analysis of the physical and chemical properties of these EPSs revealed that their molecular weight and monosaccharide composition differed from the previously reported EPSs. The EPSs synthesized by *E. aerogenes* NJ1023 could protect the engineered *E. coli* from cadaverine stress, indicating that the EPSs could be used as a novel cell protective agent. The present study has certain significance for expanding the application of *E. aerogenes* and developing novel microbial polysaccharides.

## Data availability statement

The raw data supporting the conclusions of this article will be made available by the authors, without undue reservation.

## Author contributions

YX: data curation and analysis, investigation, figure preparation, and writing. ZY: data curation and analysis, investigation, and methodology. XW: data curation and analysis. HD and WS: investigation. XH: conceptualization, funding acquisition, methodology, and writing- review and editing. KC: funding acquisition and methodology. All authors contributed to the article and approved the submitted version.

## Funding

This work was supported by the National Natural Science Foundation of China (Grant No. U21B2097), the National Key Research and Development Program of China (Grant No. 2018YFA0901500), and the Jiangsu Postdoctoral Research Foundation (Grant No. 2019 K242).

## Conflict of interest

The authors declare that the research was conducted in the absence of any commercial or financial relationships that could be construed as a potential conflict of interest.

## Publisher’s note

All claims expressed in this article are solely those of the authors and do not necessarily represent those of their affiliated organizations, or those of the publisher, the editors and the reviewers. Any product that may be evaluated in this article, or claim that may be made by its manufacturer, is not guaranteed or endorsed by the publisher.
